# The impact of mindfulness-based stress reduction on psychological health among patients with chronic diseases during COVID-19 outbreak lockdown

**DOI:** 10.1186/s43088-023-00389-2

**Published:** 2023-05-20

**Authors:** Hasnaa Osama, Hoda M. Rabea, Mona A. Abdelrahman

**Affiliations:** 1 Clinical Pharmacy Department, Faculty of Pharmacy, Beni-Suef University, Beni-Suef, Egypt; 2 Clinical Pharmacy Department, Faculty of Pharmacy, Fayoum University, Fayoum, Egypt; 3 Clinical Pharmacy Department, Faculty of Pharmacy, Nahda University, Beni-Suef, Egypt

**Keywords:** Mindfulness, MBSR, COVID-19, Chronic diseases, Psychologic distress

## Abstract

**Background:**

The emergence of COVID-19 has spurred a wide range of psychological morbidities. However, its influence on a vulnerable population with chronic conditions is less addressed. Therefore, this study aimed to investigate the psychological health among patients with chronic diseases during the elevated psychiatric distress associated with the outbreak and examine the efficacy and feasibility of mindfulness-based stress reduction intervention (MBSR). The study involved 149 participants recruited from university hospital outpatient clinics. Patients were allocated into two groups: MBSR training program and control group. Standardized questionnaires were administered to assess depression, anxiety and stress prior to the MBSR program and at completion of the training after 8 weeks.

**Results:**

The results showed that MBSR intervention improved psychological distress and decreased the mean scores of depression, anxiety and stress.

**Conclusions:**

Mindfulness training program based on audio and smartphone was feasible and effective when it was applied to patients with chronic diseases and showed positive impact on negative psychological stress domains. These findings pave the way for the integration of psychological support for patients with chronic illnesses in clinical settings.

## Background

The emergence of the novel infectious outbreak of coronavirus (COVID-19) in December 2019 is creating an unstable and rapidly changing situation [1]. When compared to the severe acute respiratory syndrome (SARS-CoV) infectious outbreak in 2003, the pneumonia outbreak associated with COVID-19 is considered as the largest so far [2]. People at risk of infection with progressively worse outcomes are elderly or those with chronic medical comorbidities. The prognosis of COVID-19 infection in severe cases can result in respiratory failure, sepsis, septic shock and death [3].

Since the identification of this outbreak and the escalating numbers of cases on a global scale, many governments, including the Egyptian government, applied a series of exceptional measures [4, 5]. These measures was adopted to control the virus transmission and to limit the infected people from being in contact with others in public places, including travel restriction and people quarantine at home, especially in the highly epidemic areas [4, 6]. Community quarantine has been introduced centuries ago as a measure to control infectious outbreaks such as cholera, SARS-CoV and Middle East respiratory syndrome (MERS-CoV) [7]. These measures, regardless of its usefulness in controlling the emerged outbreak, have restricted people’s social activities with inevitable psychological morbidities [1, 5]. Several research studies revealed a profound psychological impact and mental illness including fear due to the tremendous increase in the number of confirmed new cases and the limited knowledge of COVID-19; in addition to fear of stigma, discrimination and despair, all are well known with its association with depression and anxiety among those people [5].

Patients with chronic medical illness (e.g., chronic obstructive pulmonary disease (COPD), diabetes, heart disease and stroke) are in great risk since depression and psychological morbidities increase the burden of diseases in those patients with decrements in their quality of life, consequently affecting their chronic medical condition [3, 8, 9]. Generally, at least half of the population has one chronic disease and about 30% have two or more chronic comorbid condition [10, 11]. Compared to the public, patients with chronic diseases showed higher rates of psychological abnormalities during the pandemic [12, 13].

Although many studies represented the psychological impact of COVID-19 pandemic in general population, there is a paucity of information about the psychological distress and depression incidence and its appropriate interventions among patients with chronic comorbidities. These available interventions include counseling, support groups. Recently, cognitive-based therapeutic approaches such as mindfulness program captured an increasing interest which refers to a state of mental awareness of the present moment [14]. Mindfulness is a behavioral therapy by applying a group of exercises which mainly focus on controlling reactivity to challenging events and experiences through sensory, cognitive and emotional self-awareness. Most studies focused on the benefits of in-class mindfulness courses. Several studies and critical reviews of mindfulness efficacy evidence are encouraging with possible clinical implications to reduce psychological distress and improving health-related quality of life. Recently, instead of the traditional MBSR courses, replicated studies assessed the substantial benefits of smartphone-based mindfulness training courses. This approach would be especially of importance in circumstances of pandemic and social distancing. It also would offer a portable, low-cost and scalable format of the program [15].

Therefore, the objective of the present study was to assess the impact of the novel COVID-19 pandemic and quarantine experience on the psychological stress and mental health levels and to examine mindfulness training program efficacy and feasibility. The assessment included depression and anxiety severity and prevalence in population with diagnosed chronic diseases.

## Methods

### Study design and population

A cross-sectional survey was designed to assess the psychological response and mental health during COVID-19 epidemic by using a questionnaire among patients with chronic diseases. Patients were enrolled from outpatient clinics at Beni-Suef University hospital in Beni-Suef, Egypt. Responses were collected from March 12th, 2020, to June 19th, 2020.

After the acceptance of the respondents using an informed consent, they filled out the demographic details. Then the participants were asked to answer a series of several questions, consecutively collecting information on knowledge about COVID-19, the precautionary measures implemented recently against COVID-19 and the psychological impact and mental health behaviors among patients with chronic disease residents. The study conductance was approved by the University Institutional Ethics Research Committee.

### Inclusion and exclusion criteria

Egyptians of both genders with a documented chronic disease were included. Chronic diseases were defined as persistent or long-lasting medical conditions, typically more than 1 year that require ongoing medications and monitoring [16].

A checklist included the most common chronic diseases with potentially a wide range of health problems such as myocardial infarction, type 2 diabetes mellitus or hypertension was used. All of the included participants were cooperative and willing to fill informed consent. Patients with severe chronic disabling diseases that would restrain or limit the patient’s ability to respond to the questionnaire or adequately participate in the daily required activities such as cancer, epilepsy or any intellectual or psychiatric illness were excluded.

### Data collection

The collected sociodemographic data included age, gender, marital and parental status, educational attainment and employment status. Participants were also asked to generally rate their physical health status illness. Other clinical data including patient’s history and medical records were collected. History of close contact with individual with confirmed or suspected COVID-19 infection or suspected materials was documented.

Knowledge questionnaire consisted of 10 questions about COVID-19 epidemic were developed by authors. The questionnaire included questions regarding the possible routes of transmission, methods of diagnosis and the main clinical symptoms. Participants were asked to elucidate their most common source of information and to express their degree of concern about self or any other family member getting infected and the chance of survival in case of infection. The answers for these questions were divided into “Agree,” “Disagree” or “I don’t know.” The points for each correct answer were calculated on the basis of 1 point for answer “Agree” and 0 points for either “Disagree” or “I don’t know” with a total score of knowledge ranged from 0 to 10. The internal consistency of the developed knowledge questionnaire was 0.77 as estimated by the coefficient of Cronbach’s alpha.

The psychological distress was assessed using the Impact of Event Scale-Revised (IES-R) questionnaire. The participants self-completed the questionnaire that has been previously validated in Arabic to estimate the psychological impact of COVID-19 epidemic [3, 13, 17]. The IES-R questionnaire consisted of 22 variables with three subscales to estimate the average avoidance, intrusion and hyperarousal [3]. The overall score of IES-R was divided normal for scores up to 23, a mild psychological effect for scores ranged from 24 to 32, a moderate psychological impact for scores ranged from 33 to 36 and severe psychological effect for scores higher than 37 [18].

For mental health assessment, Depression, Anxiety and Stress Scale (DASS-21) were used which is a short version of the original DASS questionnaire. Therefore, to establish a reliable cutoff values with relative severity, the scores of DASS-21 were doubled. The outcomes of DASS-21 scoring system for depression, anxiety and stress were classified into normal, mild, moderate and extremely severe according to Henry and Crawford, 2005 [19, 20]. The depression subscale calculation was classified according to scores as follows: normal (0–9), mild (10–13), moderate (14–20), severe (21–27) and extremely severe depression (> 28). The anxiety subscale scoring was divided according to scores into normal (up to 7), mild (8–9), moderate (10–14), severe (15–19) and extremely severe anxiety (> 20). The overall stress score was divided into five subdivisions including normal, mild, moderate, severe and extremely severe stress with score ranges from 0 to 14, 15–18, 19–25, 26–33 and > 34, respectively. For this study, validation using Cronbach’s alphas resulted in reliable coefficients of the scale with values of 0.78, 0.82 and 0.83 for the DASS-21 subscales: Anxiety, Depression and Stress, respectively.

### Assessment of the mindfulness stress management program

**Fig. 1:**
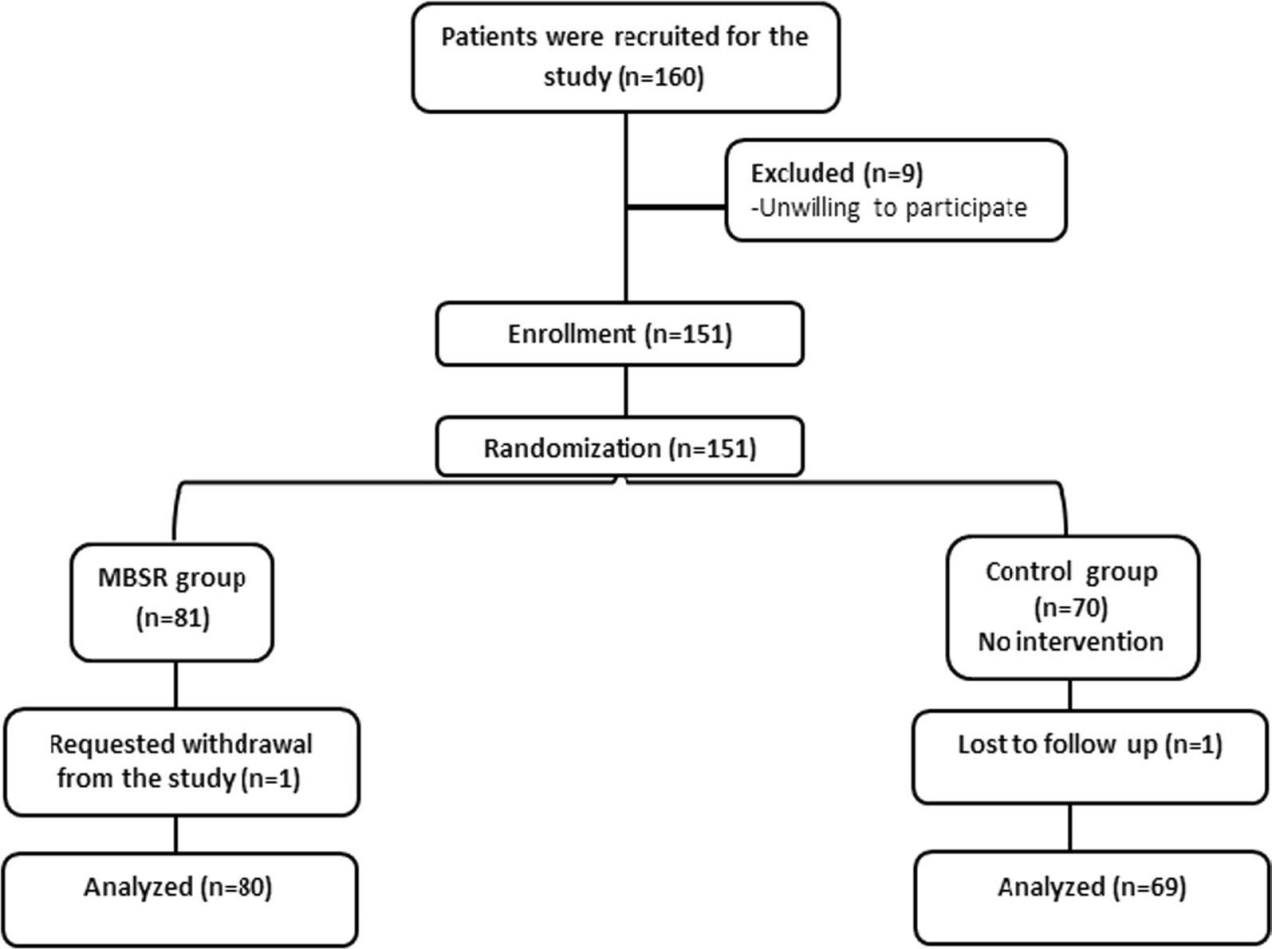
The CONSORT flowchart of the study

The recruited participants were randomized into two groups: the intervention group (*n* = 80), which received MBSR training, and the control group (*n* = 69) without intervention. Both groups were matched in terms of the baseline psychological scales and demographic characteristics. Figure 1 illustrates the CONSORT flowchart of the study. The efficacy of a mindfulness-based stress reduction (MBSR) course was estimated by implementing 8-week trial using 90 min of audio in a compact CD of the mindfulness practices in a daily routine and meditation practices guided by smartphone applications given. Healthy Minds Program smartphone application was used for further guided practices and video illustrations. All participants were given information and trained about mindfulness techniques by an MBSR instructor with an experience in meditation practice. Initially, an introduction about the main concept of the mindfulness techniques and understanding the impact of psychological distress management were given by the researchers. Mindfulness practice aimed to increase awareness of present moment experiences which linked to greater well-being and benefits for health and psychology.

The program included different breathing and meditation exercises, including observing, describing, acceptance, breathing awareness and body scanning. The sessions in the eight weeks were designed to improve the participant’s self-monitoring and control. The sessions also included meditation and mindful-body examination, 3-min conscious seeing and hearing practice, breathing exercises and home works. To ensure adherence and flexibility, the participants choose by themselves the time and space for mindfulness practices each day. Diary records and practice sheets were used for daily assessment and to ascertain adherence to the applied program throughout the trial duration [21]. Text messages and phone calls were used to remind the participants about the required activities and to resolve any technical problems or receive participants’ questions. Participants were awarded health insurance benefits for each week training completion. Psychological measures using IES-R and DASS-21 were evaluated before and after the intervention.

### Statistical analysis

The collected variables including demographic characteristics and knowledge variables were statistically analyzed using IBM software SPSS 16.0 (Install Shield Corporation, Inc., Chicago, IL, USA). The percentages of the collected responses with respect to the total number of responses for a question were calculated and tabulated. The normally distributed scores of the IES-R and DASS subscales were expressed by mean and Standards deviation. Multivariable linear regression analysis was conducted to identify the associations between all of the collected variables. Independent student t test, ANOVA test and Chi-square tests were applied and the statistical significance level for all tests was set at *p*-value of < 0.05, (Two-tailed).

## Results

### Sociodemographic characteristics and knowledge about COVID-19 outbreak

In the time frame from 12th April to 19th June of 2020, 160 patients were recruited to participate in the present study. Only 149 participants completed the study and included104 (69.7%) males and 45 (30.2%) females. The withdrawn eleven patients did not complete their questionnaire or unwilling to continue the 8-week mindfulness intervention practice. The demographic and prevalence of chronic diseases among the study group are described in Table 1.

**Table 1:** Clinical and demographic characteristics of the participants and their average knowledge scores (*n* = 149)

Variable	Number of participants (%)	Knowledge score (mean ± standard deviation)	*p*-value
Age mean (SD)	45.67 (9.92)	7.073 ± 1.89	
*Gender*			
Male *n* (%)	104 (69.7%)	6.99 ± 1.57	0.602
Female *n* (%)	45 (30.2%)	7.15 ± 2.01	
*Marital status*			
Married	110 (73.8%)	7.13 ± 1.78	0.327
Not married	32 (21.5%)	6.89 ± 1.93	
Divorced/separated	7 (4.7%)	6.14 ± 1.31	
*Education level*			
None	2 (1.34)	3 ± 0.011	< 0.001
High school	3 (2.01)	6.15 ± 0.03	
University	108 (72.4)	6.43 ± 1.33	
University and Master or Doctorate)	36 (24.16)	9.35 ± 0.63	
*Child(ren) in house*			
Yes	95 (63.7)	7.05 ± 1.72	0.847
No	54 (36.2)	7.11 ± 1.98	
*The prevalence of chronic diseases among participants*			
Asthma	3 (2.01)	6.8 ± 2.04	0.839
Diabetes mellitus	48 (32.2)	7.06 ± 1.85	
Hypertension	54 (36.24)	7.15 ± 1.81	
Cardiovascular diseases	8 (5.36)	7.5 ± 1.77	
Liver diseases	20 (13.42)	7.13 ± 1.36	
Kidney diseases	16 (10.74)	6.57 ± 1.24	

The mean age of the recruited participants was 45.67 ± 9.92 and ranged between 28 and 65 years. Most of the participants held a university degree (*n* = 108, 72.48%) and were married (*n* = 110, 73.8%).

The average knowledge score was 7.073 ± 1.89 and ranged between 3 and 10. The estimated knowledge scores showed a significant difference across education levels (*p* < 0.001). However, the categories of gender and marital status showed insignificant difference in knowledge scores; 0.602 and 0.327, *p* > 0.05, respectively (Table 1). The linear regression analysis revealed that high education levels were significantly associated with high knowledge scores, β: 2.52, (B = 0.741, 95% CI 2.14–2.89, *p* < 0.001). Regarding the other sociodemographic characteristics of the participants including age, marital status and parental status, there were no associations with knowledge scores.

### The psychological impact association with the sociodemographic variables among *participants*

The psychological impact as interpreted from the DASS-21 and IES scoring systems during COVID-19 outbreak and its association with the sociodemographic characteristics of the participants was analyzed using multiple regression of IES, and DASS-21 scores as independent variables whereas age, marital status, education degree and chronic disease status were considered as factors (Table 2). The effect of chronic diseases on psychological distress was estimated in all participants. Chronic diseases were highly associated psychological distress, being highest for liver and renal diseases followed by cardiovascular diseases, asthma, hypertension and diabetes, respectively.

**Table 2:** Estimation of the association between sociodemographic variables and psychological scoring systems including IES-R and DASS-21 during the COVID-19 outbreak (bold font indicates statistical significance)

Variables		IES			Depression			Anxiety			Stress		
		Exp. B	95% CI	*p*-value	Exp. B	95% CI	*p*-value	Exp. B	95% CI	*p*-value	Exp. B	95% CI	*p*-value
Age mean (SD)	45.67 (9.92)	0.104	− 0.028 to 0.131	0.201	− **0.152**	− **0.098 to** − **0.005**	**0.029**	**0.71**	− **0.063 to** − **0.011**	**0.001**	**0.152**	− **0.037 to** − **0.011**	**0.029**
*Gender*													
Male no. (%)	104 (69.7)	− **0.587**	**2.81 to 4.53**	**0.006**	**0.56**	**(0.26 to 4.35)**	**0.013**	**0.806**	**4.67 to 6.05**	**0.009**	**0.291**	**0.61 to 0.27**	** < 0.001**
Female no. (%)	45 (30.2)	**Ref.**	**Ref.**	**Ref.**	**Ref.**	**Ref.**	**Ref.**	**Ref.**	**Ref.**	**Ref.**	**Ref.**	**Ref.**	
*Marital status*													
Married (%)	110 (73.8)	**0.085**	**1.33 to 1.50**	**0.043**	0.725	− 1.28 to 0.478	0.371	0.908	− 1.17 to 0.75	0.591	**0.714**	**1.42 to 0.68**	**0.038**
Not married (%)	32 (21.5)	− 0.063	− 1.56 to 1.97	0.173	1.27	− 0.987 to 1.64	0.691	0.801	− 1.51 to 0.57	0.690	0.782	− 1.93 to 0.77	0.449
Divorced or separated (%)	7 (4.7)	**Ref.**	**Ref.**	**Ref.**	**Ref.**	**Ref.**	**Ref.**	**Ref.**	**Ref.**	**Ref.**	**Ref.**	**Ref.**	
*Education level*													
None (%)	2 (1.34)	**Ref.**	**Ref.**	**Ref.**	**Ref.**	**Ref.**	**Ref.**	**Ref.**	**Ref.**	**Ref.**	**Ref.**	**Ref.**	
High school (%)	3 (2.01)	**0.041**	**0.022 to 0.83**	**0.043**	**0.781**	− **0.455 to** − **0.131**	**0.002**	0.791	− 0.35 to 0.07	0.165	0.946	− 0.20 to 0.05	0.237
University (%)	108 (72.4)	**0.271**	**0.264 to 0.87**	**0.016**	0.877	− 0.814 to 0.552	0.707	0.628	− 0.43 to 0.61	0.371	0.922	− 0.32 to 0.13	0.317
Graduate with MSc. or PhD.)	36 (24.16)	0.261	− 0.54 to 1.32	0.41	0.849	− 0.531 to 0.43	0.641	1.542	0.18 to 0.97	0.024	1.242	− 0.15 to 0.59	0.258
*Child(ren) in house*													
Yes (%)	95 (63.7)	1.64	− 0.35 to 1.16	0.31	1.842	− 0.036 to 0.895	0.29	0.941	− 0.49 to 0.36	0.173	1.244	− 0.36 to 0.58	0.47
No (%)	54 (36.2)	**Ref.**	**Ref.**	**Ref.**	**Ref.**	**Ref.**	**Ref.**	**Ref.**	**Ref.**	**Ref.**	**Ref.**	**Ref.**	
*Chronic diseases among participants*													
Asthma (%)	3 (2.01)	**Ref.**	**Ref.**	**Ref.**	**Ref.**	**Ref.**	**Ref.**	**Ref.**	**Ref.**	**Ref.**	**Ref.**	**Ref.**	0.183
DM (%)	48 (32.2)	1.489	− 0.325 to 4.27	0.183	0.087	− 0.163 to 0.204	0.733	**.**0.708	− 1.56 to 0.83	0.565	0.582	− 1.68 to 0.56	0.331
HTN (%)	54 (36.24)	0.622	− 1.57 to 4.64	0.401	0.931	− 0.003 to 0.163	0.053	1.031	− 1.255 to 1.84	0.063	0.902	− 0.33 to 0.79	0.485
Heart diseases (%)	8 (5.36)	0.325	− 0.45 to 1.83	0.062	0.780	− 0.476 to 0.079	0.149	0.811	− 1.39 to 0.86	0.590	1.733	− 0.14 to 1.25	0.123
Liver diseases (%)	20 (13.42)	**2.55**	**10.68 to 16.73**	**0.002**	**1.719**	− **0.68 to** − **0.015**	**0.043**	**1.821**	**0.03 to 0.772**	**0.023**	**1.605**	**0.036 to 0.49**	**0.018**
Kidney diseases (%)	16 (10.74)	**1.143**	**7.11 to 10.24**	**0.016**	**2.051**	**0.79 to 1.461**	** < 0.001**	**1.432**	**0.11 to 0.69**	**0.006**	**0.974**	**0.012 to 0.47**	**0.041**

#### The Impact of Event Scale-Revised association with demographic variables

Men showed a significantly lower scores of IES-R (B = − 0.587, 95% CI 2.81 to 4.53); however, high school and university students showed a significant higher IES-R (B = 0.426, 95% CI 0.022–0.83) and (B = 0.57, 95% CI 0.264–0.87), respectively, compared to uneducated participants and graduates. The estimated R2 (Nagelkerke’s pseudo R2) of 0.098 indicated that about 10% of the variability is explained by the significant demographic variables.

#### DASS-21 scores association with demographic variables

DASS depression subscale scores were in the moderate levels in about 43.6% (*n* = 65) of participants, while those with high scores of depression were 21.5% (*n* = 32). Only 18.1% (*n* = 27) were in the extremely high range. Male gender with liver or kidney diseases lower grades of education were associated with highly significant depression levels.

Of the 149 participants, about 54% were in the moderate levels of anxiety subscale, while high and extremely high scores of anxiety were found in 24% and 12.8%, respectively. Male gender showed higher anxiety levels. Regarding stress subscale, about 48.3% (*n* = 72) of participants were in the moderate levels, 23.48% (*n* = 35) were in the high levels, and 24.8% (*n* = 37) were in the extremely high stress range. Higher scores of stress subscale were associated significantly with male gender (B = 0.291, 95% CI 0.61–0.27, *p* < 0.001), married (B = 0.714, 95% CI 1.42 to 0.68) and liver and kidney chronic diseases (B = 1.605, 95% CI 0.036–0.49) and (B = 0.974, 95% CI 0.012–0.47), respectively.

### Mindfulness-based stress reduction (MBSR) training course

**Fig. 2:**
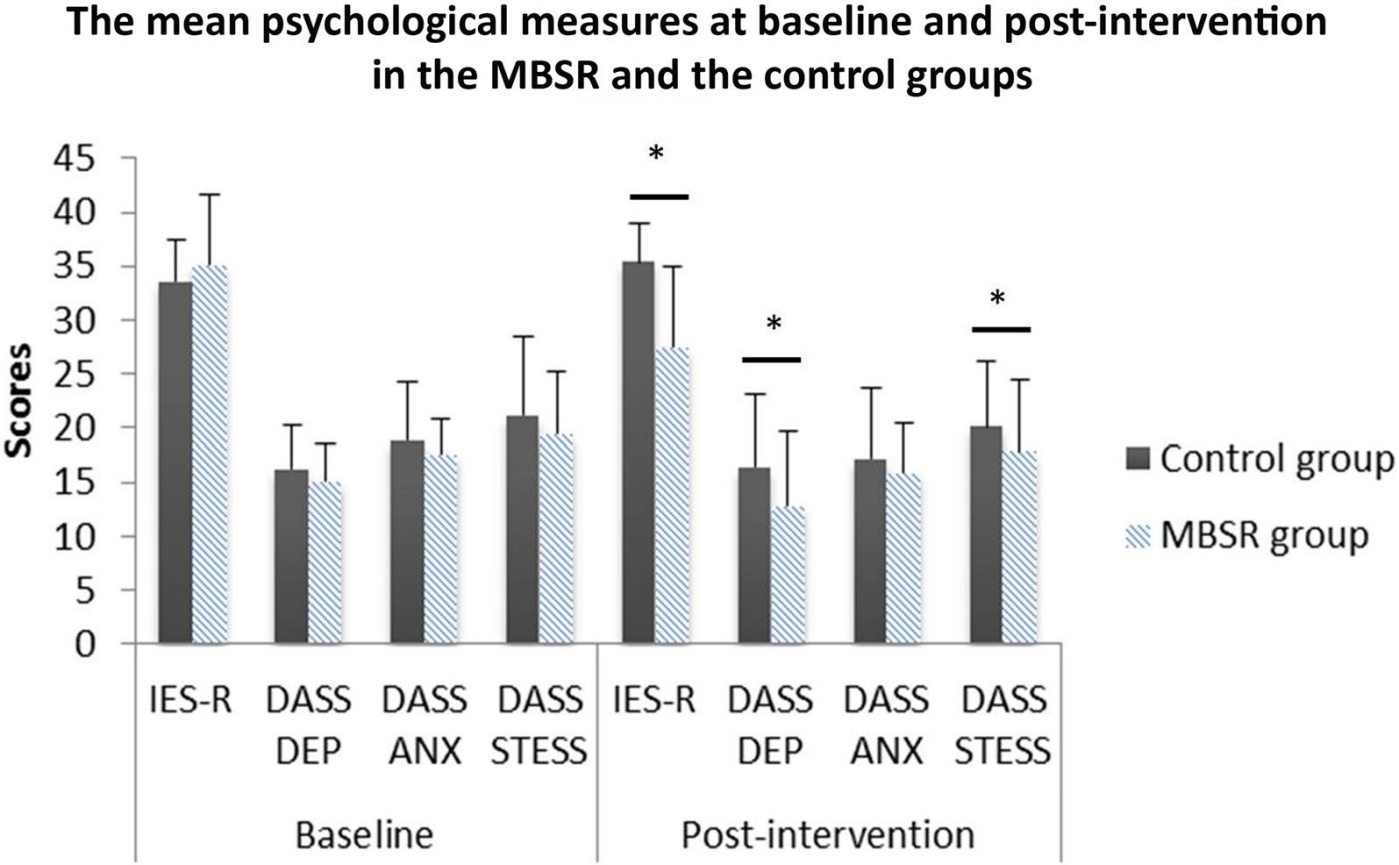
The mean psychological measures between the mindfulness-based stress reduction intervention (MBSR) and control group at baseline and post-intervention. DASS-ANX: DASS anxiety

At baseline, the MBSR and the control group were well matched with a non-significant difference in IES-R (*p*-value = 0.068), and DASS-21 scores; *p*-value = 0.083, 0.079 and 0.102, for depression, anxiety and stress, respectively. Table 3 displays the results of data analysis before and after the program. The difference in the psychological scales mean values in MBSR was statistically significant in IES-R, DASS-21 depression and stress components as compared with the control group with estimated *p* < 0.001, 0.002 and 0.032, respectively (Fig. 2). Moreover, the control group showed a trend of increase in the IES-R and DASS-21 scores. After mindfulness training, IES-R scores were decreased by 7.75 points and the difference was statistically significant (*p* < 0.001). Also, DASS-21 depression subscale was decreased after the training program and the difference was statistically significant (*p* = 0.011). Despite the non-significant difference in anxiety and stress subscales (*p* = 0.071, 0.138, respectively) after the mindfulness training from baseline, a noteworthy trend of reduction was observed.

**Table 3:** Descriptive Statistics of IES-R and DASS-21 scale at baseline and after mindfulness-based intervention (MBI) (*n* = 80)

Variables	Before MBSR	After MBSR	*p*-value
	Mean (SD)	Mean (SD)	
	(Min–max)	(Min–max)	
IES-R	35.17 (3.9)	27.42 (3.61)	< 0.001*
	(22–38)	(19–31)	
DASS-21 depression subscale	15.1 (4.12)	12.82 (6.73)	0.011*
	(9–30)	(7–22)	
DASS-21 anxiety subscale	17.6 (5.34)	15.9 (6.42)	0.071
	(9–23)	(8–16)	
DASS-21 stress subscale	19.41 (7.21)	17.83 (6.15)	0.138
	(13–34)	(6–19)

## Discussion

Psychological anxiety and stress concerns socially affect each individual to different degrees worldwide during the period of the COVID-19 pandemic [5, 22]. Therefore, the present study aimed to determine the psychological impact of COVID-19 outbreak on Egyptian adults with chronic diseases and to relate the benefits and efficacy of MBSR training program in clinical settings. Since the spread of the virus is ongoing with increasing numbers of cases and concerning fatality, several studies underline a considerable prevalence of psychological distress among the population. However, those with chronic diseases are at a considerable risk of having a higher psychological burden due to the identified risk of severe or unfavorable course of respiratory distress manifestations with COVID-19, in addition to the specter of targeted medical support and treatment shortage [10, 23].

We enrolled 149 patients in the study with different chronic diseases including hypertension, diabetes, heart, liver, kidney and pulmonary-related diseases. About 21% of participants had more than one chronic disease. The psychological impact of the pandemic was investigated using IES-R and DASS-21 scoring systems at baseline and after eight weeks for the mindfulness program interventional group and the control group.

The results of the present study revealed that patients with chronic diseases had high rates of psychological distress with IES-R score mean value of 35.17, where about 66.3% of participants were in moderate and severe range, especially for those with liver or renal disorders compared to other chronic diseases included in the study, in addition to high prevalence of depression, anxiety and stress as estimated by the DASS-21 scoring system with a mean value of 15.1, 17.6 and 19.4, respectively. These findings conform to several psychological research studies which identified having a chronic condition as a positive predictor for the score of impact of event, depression, stress and anxiety with a wide variability depending on the study population and diagnostic tools [3, 5, 22, 23]. The burden of COVID-19 pandemic on the healthcare systems adversely affected patients with chronic diseases and their regular routine for disease management and follow-up. Consequently, the psychological health was also affected for those patients [12, 13].

The assessment of psychological state after mindfulness intervention program showed a potential significant efficacy in improving psychological distress, depression and stress in those patients after the first lockdown when compared to the control groups. Moreover, the preliminary findings of the present study demonstrated a significant worsening in the psychological measures in the control group. Mindfulness-based interventional approaches have grown evidence of efficacy with widespread acceptance [24]. The present study takes precedence in implementing MBSR training in a cultural context that differed from its original culture and spiritual ideas from which it was derived.

Educating mindfulness can also help patients in adjusting better to chronic incurable diseases through reduction of mood disturbances. Furthermore, MBSR can help as a complementary strategy to deal with emotions, negative thoughts, enhance self-awareness and experience positive mental events [14, 25]. Therefore, MBSR benefits are outlined as the 3rd wave of behavioral treatment for incurable illnesses.

A study conducted by Nyklíček et al. [26] investigated the effect of mindfulness stress reduction program on over a hundred participants with coronary diseases and concluded that a brief MBSR training program of three 90–120 min sessions per week was effective to improve psychological distress which is in support of the present study findings. Another study revealed that MBSR course practice for 2.5 h would be effective to reduce stress and depression significantly among patients with chronic illness [27].

However, studies conducted using shorter length of intervention (30 min for 7 days) reported improvement in anxiety without significant improvement in depression [28]. Also, Song and Lindquist (2015) claimed that the duration of at least 90 min/session should be performed in mindfulness program to achieve significant impact on psychological disturbances including depression, anxiety and stress which is supported by our study findings. Therefore, standardization of the length of MBSR training course and frequency of sessions should be established to gain positive psychological effects [29].

While the majority of the literature has focused on the benefits of group-based mindfulness programs, mindfulness programs based on smartphone applications offer more feasibility and training benefits regardless of time or space barriers that could hinder in-person classes. This digital approach also applies in special circumstances, such as during the pandemic social distancing and the lockdown because of safety concerns [30, 31].

To sum up, our findings will be beneficial to provide vital guidance for better development of psychological support for patients with chronic diseases by consideration of psychological education and interventions such as cognitive behavior therapy (CBT) either online or using smartphone applications in health care settings [32].

Although the findings of the present study emphasize the positive psychological outcomes after the application of MBSR program, there are several limitations. Firstly, this study is devoid of post-intervention follow-up for participants to assess the potential long-term efficacy of MBSR training course. Secondly, the involved sample size is relatively small in addition to dependence on questionnaire of the outcomes without clinical assessment. Thirdly, the study design lacks the presence of active control group and is based on self-report assessment measures. Another limitation of this study is the relatively small number participating females, hence preventing the examination of gender effect. Large randomized controlled trials (RCTs) with long-term follow-up, and a standardized set of output measures, should be considered in future investigations metrics to assess the efficacy of MBSR in enhancing mental and physical well-being among chronic-diseased patients.

## Conclusions

The era of COVID-19 epidemic has a significant psychological impact on general population, especially those with incurable diseases. This study revealed that MBSR program is feasible non-pharmacological approach that could have salutary effect on stress and depression in patients with chronic diseases and it is desirable to be considered in specialized clinics of chronic disorders. Future larger controlled studies with proper participants’ stratifications are needed to further examine the potential clinical benefits of the overall approach.

## Data Availability

The datasets used and/or analyzed during the current study are available from the corresponding author on reasonable request.

## Abbreviations

CBT: Cognitive behavior therapy
DASS-21: Depression, Anxiety and Stress Scale
IES-R: Impact of Event Scale-Revised
MBSR: Mindfulness-based stress reduction intervention
RCTs: Randomized controlled trials
